# Enhancement of multitasking performance and neural oscillations by transcranial alternating current stimulation

**DOI:** 10.1371/journal.pone.0178579

**Published:** 2017-05-31

**Authors:** Wan-Yu Hsu, Theodore P. Zanto, Martine R. van Schouwenburg, Adam Gazzaley

**Affiliations:** 1 Department of Neurology, University of California San Francisco, San Francisco, California, United States of America; 2 Neuroscape, University of California San Francisco, San Francisco, California, United States of America; 3 Department of Psychiatry, University of California San Francisco, San Francisco, California, United States of America; 4 Department of Physiology, University of California San Francisco, San Francisco, California, United States of America; University Medical Center Goettingen, GERMANY

## Abstract

Multitasking is associated with the generation of stimulus-locked theta (4–7 Hz) oscillations arising from prefrontal cortex (PFC). Transcranial alternating current stimulation (tACS) is a non-invasive brain stimulation technique that influences endogenous brain oscillations. Here, we investigate whether applying alternating current stimulation within the theta frequency band would affect multitasking performance, and explore tACS effects on neurophysiological measures. Brief runs of bilateral PFC theta-tACS were applied while participants were engaged in a multitasking paradigm accompanied by electroencephalography (EEG) data collection. Unlike an active control group, a tACS stimulation group showed enhancement of multitasking performance after a 90-minute session (F_1,35_ = 6.63, p = 0.01, ηp^2^ = 0.16; effect size = 0.96), coupled with significant modulation of posterior beta (13–30 Hz) activities (F_1,32_ = 7.66, p = 0.009, ηp^2^ = 0.19; effect size = 0.96). Across participant regression analyses indicated that those participants with greater increases in frontal theta, alpha and beta oscillations exhibited greater multitasking performance improvements. These results indicate frontal theta-tACS generates benefits on multitasking performance accompanied by widespread neuronal oscillatory changes, and suggests that future tACS studies with extended treatments are worth exploring as promising tools for cognitive enhancement.

## Introduction

Engaging in two or more cognitive processes simultaneously is a common demand in today’s saturated, information-rich environment. Although processing multiple streams of content at the same time is not beyond human ability, managing overlapping attention-demanding tasks diminishes performance [1]. Optimal neural processing engaged during high-level cognitive performance has been shown to rely on cortical oscillatory synchronization in different frequency bands [2, 3], such as theta (4–7 Hz) oscillations in working memory [4], retrospective monitoring [5], and focused attention [6]. Notably, prefrontal cortical theta oscillations have been associated with multitasking performance [7, 8]. Based on this finding and evidence that transcranial direct current electrical stimulation (tDCS) can exert beneficial effects on multitasking performance [9, 10], here, we investigate whether applying an alternating electrical stimulation at theta oscillations across the prefrontal cortex (PFC) would similarly improve performance metrics of multitasking.

Transcranial alternating current stimulation (tACS) is a non-invasive brain stimulation technique that modulates brain activity via weak sinusoidal electrical stimulation. The electrical currents generated by tACS are not constant (i.e. direct current) but alternate between stimulation electrodes in a frequency-specific manner. In line with the perspective that tACS is capable of influencing with endogenous brain oscillations and brain functions [11–13], numerous behavioral studies have revealed that tACS affects motor function [14–16], perception [17–19], and higher-order cognitive function [20, 21], in many cases making a correlational links between brain oscillations and behavior [5, 22, 23]. However, the investigations of the neurophysiological effects of tACS have been limited [24–26]. While some studies have demonstrated that tACS enhances power of endogenous brain oscillations in a frequency-specific manner [27–29], other studies have shown evidence that tACS alters power of neuronal oscillations in frequencies other than the stimulating frequency (i.e. cross-frequency modulation) [30–32].

In the present study, we aimed to assess whether applying bilateral PFC theta-tACS would improve performance metrics of multitasking, and explore the associated changes in electrophysiological measures. To accomplish this, brief repetitive runs of tACS were delivered over each participant’s bilateral PFC (at electrode locations F3 and F4) while electroencephalography (EEG) data was acquired during control runs. In addition, an active control group received very brief durations of tACS, which have proven ineffective in inducing changes in other studies [23, 31, 33]. During all runs, participants were engaged in a cognitive paradigm in the form of a video game (NeuroRacer) [7] designed to challenge and assess multitasking performance by measuring perceptual discrimination (d’) in the context of simultaneous visuomotor tracking.

## Materials and methods

### Participants

Forty healthy adults (mean age: 26.3 y/o; range 18–35 years; 19 males) gave informed consent to participate in the study according to procedures approved by the Committee for Human Research at the University of California at San Francisco. All participants had normal or corrected-to-normal vision, and were free from neurological/psychiatric disorders or other contra-indications for tACS exposure.

Participants were randomly assigned into the tACS group (mean age: 26.7 y/o; 10 males) or a control group (mean age: 25.8 y/o; 9 males). Two participants’ data were excluded from the analyses (one participant in the tACS group dropped out after the thresholding, prior to tACS, due to a personal reason, and one participant in the control group performed more than 2.5 standard deviations above the mean of the two groups, indicating the thresholding was insufficient). Of the remaining 38 participants, three participants’ EEG data were excluded (two from the tACS stimulation group, one from the control group) from the analyses due to excessive noise.

### Experimental procedure

**Fig 1:**
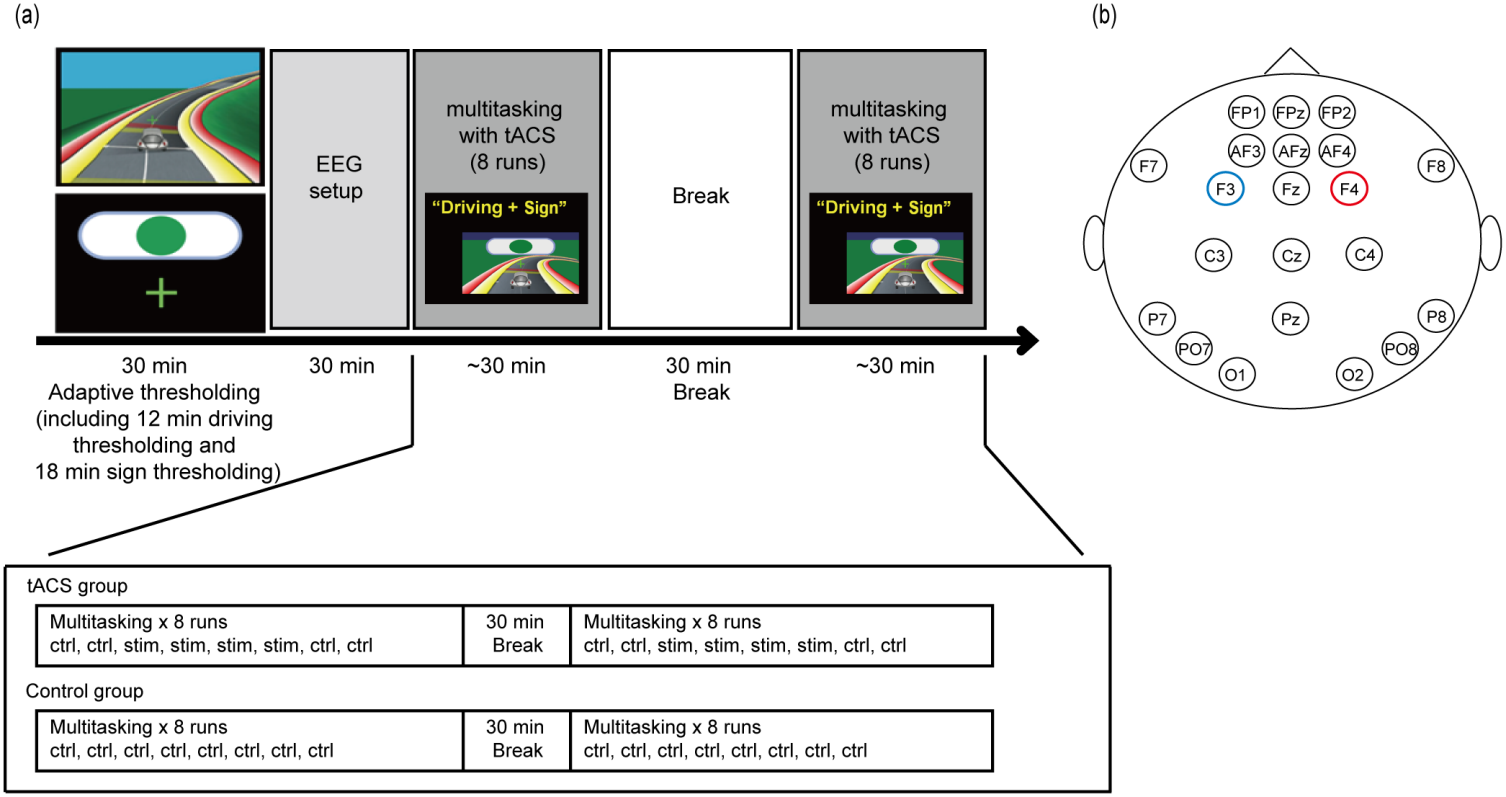
NeuroRacer and experimental design. (a) Experimental procedure (b) A 6-Hz sinusoidal alternating current of 1000uA was delivered to F3 and F4 with a 180 degree phase offset. stim, tACS stimulation run; ctrl, control stimulation run.

Fig 1a shows the experimental procedure. At the beginning of the study, an adaptive thresholding procedure was conducted (see experimental paradigm section below). This was followed by the EEG/tACS setup procedure and sixteen 3-min multitasking runs (perform the sign task and driving task concurrently) with a 30-min break after the 8th run. Participants in tACS stimulation group received stimulation in third, fourth, fifth, sixth, eleventh, twelfth, thirteen and fourteenth runs and control stimulation for all other runs. The active control group received only control stimulations (see transcranial alternating current stimulation part below) for all 16 multitasking task runs (Fig 1a).

### Experimental paradigm

NeuroRacer software was developed using the OpenGL Utility Toolkit (GLUT; http://www.opengl.org/resources/libraries/glut/) as a video game that assesses multitasking performance by challenging visual discrimination ability (sign task) in the context of visuomotor tracking (driving task) [7]. The visuomotor tracking (driving task) required participants to control a car, keeping it in the center of the road, and avoiding the yellow and red boundaries (Fig 1a). The road was created by track pieces that included right and left turns, as well as uphill and downhill pieces. These pieces were presented pseudo-randomly for 2, 2.5 or 3 seconds, generating a path that the participant had to guide the car on. For the visual discrimination task (sign), participants were presented with targets (green circles) and non-targets (green pentagons and squares; blue and red circles, pentagons, and squares). The signs were randomly presented for 400 ms every 2, 2.5, or 3 seconds. The participants were instructed to ignore the non-target signs and selectively respond to the target sign. A fixation cross that provided performance feedback was present on the screen at all times below the signs (and above the car during the multitasking condition): it turned green for 50 ms when the participant responded to the target sign within the proper amount time after the target showed up, or when a non-target sign was correctly ignored. When either of the aforementioned conditions were not met, it would turn red for 50 ms. At the beginning of the study, an adaptive thresholding procedure of both single tasks—visuomotor tracking and visual discrimination ability—was conducted to determine a “driving” and “sign discrimination” difficulty level so that each participant would perform at ~80% accuracy (for more details, please see Methods section of Anguera et al., 2013). During the multitasking experimental runs (Fig 1a), participants were instructed to perform the sign task and driving task concurrently. Participants had to respond to the target sign as fast as possible by pressing a button on a Logitech gamepad controller (Logitech, USA) with their right thumb, while simultaneously controlling the car by using the left thumb-stick of the game controller (for more details, please see Methods section of Anguera et al., 2013).

### Transcranial alternating current stimulation (tACS)

tACS was applied at 6 Hz via a pair of Ag/AgCl electrodes (3.14 cm^2^) through a Starstim device (Neuroelectrics, Spain). The stimulation electrodes were located over bilateral PFC centered at F3 and F4 of the 10–20 electrode coordinate system (Fig 1b). A sinusoidal alternating current of 1000 uA peak amplitude (2000 uA peak-to-peak amplitude) was delivered to F3 and F4 with a 180 degree phase offset. The impedance was kept below 10 kΩ. For stimulation runs, the alternating current was delivered while participants were engaged in the NeuroRacer multitasking paradigm (3 min stimulation including 10-s ramp up and 10-s ramp down). For control runs, the 10-s ramp up period was immediately followed by a 10-s ramp down period and turned off for the remainder of the 3 min run.

In order to confirm that the control runs were an appropriately blinded manipulation, all of the participants were asked to complete a 1-min survey to scale the perception of stimulation (headache, neck pain, scalp pain, tingling, itching, burning sensation, sleepiness, trouble concentrating, and acute mood change) from 1 (mild) to 10 (severe) at the end of each run.

### Behavioral data analysis

To determine the effects of tACS on multitasking performance, we evaluated perceptual discrimination performance during each multitasking run using a metric of discrimination performance (d-prime), which was estimated for each participant by comparing hit (correct responses to target signs) rates and false alarm (responses to non-targets) rates and calculated as d’ = Z(hits)–Z(false alarms). Since all participants were receiving control stimulation during the first two runs, d-prime obtained from the first two runs were averaged to serve as baseline performance levels, i.e. pre-stimulation performance. These first two runs were matched across groups by design to provide critical evidence that the two groups exhibit comparable performance prior to applying different stimulation protocols between the groups. D-prime measured in the last two runs (i.e. runs 15 and 16) were averaged as the measure of post-stimulation performance. This approach makes the pre- and post- stimulation data comparative by ensuring these data points are equally powered. During these runs, both groups received same amount of stimulation (i.e. control runs were applied in both groups) and because stimulation did not extend throughout the run, tACS artifacts did not contaminate the EEG signal, enabling the assessment of electrophysiological activity.

### Electroencephalography data acquisition and analysis

Electrophysiological signals were recorded with a wireless, dry electrode Enobio-20 system (Neuroelectrics, Spain) with a sampling rate of 500 Hz. One of the 20 electrodes was used as an electrooculogram (EOG) channel. The remaining 19 channels were distributed over the scalp (F7, AF3, FP1, FP2, AFz, AF4, F8, P8, C4, O2, Cz, Pz, PO8, C3, C3, PO7, Fz, P7, O1) (Fig 1b). Raw EEG data were analyzed using the FieldTrip toolbox (http://fieldtrip.fcdonders.nl/ [34]). As d-prime was used as the behavioral measurement and it takes performance on every trial into account, we collapsed across all trial types (target and non-target) for subsequent EEG data analyses. Only data from the control runs when stimulation was not occurring were analyzed to prevent any potential confound introduced by tACS artifacts.

Data were segmented into epochs beginning 1000 ms before to 1000 ms post sign onset and were demeaned/detrended and referenced to the average EEG signal. An independent component analysis (ICA) was performed to remove components consistent with topographies for blinks and eye movements. An average of 5% ICA components were rejected. Epochs that exceeded a voltage threshold of 80 uV were rejected. An average of 32.0% and 28.9% trials were rejected per run for the tACS and control groups, respectively. Data across runs were then grouped in the same way as behavioral analyses, where the first two runs were averaged to serve as the pre-stimulation baseline and the last two runs (i.e. runs 15 and 16) were averaged together as the measure of post-stimulation electrophysiological activity. Time frequency analyses were conducted from 500 ms before to 600 ms after the onset of the sign presentation. As the nature of the present study was to explore effects of tACS on neurophysiological measures, a fast fourier transformation was performed from 2 to 30 Hz. The data were multiplied by a hanning taper, using a sliding time window. Power changes of oscillatory activity as a function of time were calculated for theta (4-7Hz), alpha (8-12Hz), and beta (13-30Hz) bands with one power value every 10 ms. The data was referenced to a baseline period (500 ms to 100 ms prior to the sign onset of the pre-stimulation measure) according to the following formula: Power change(%)(*k*) = (A(*k*)-R)/R*100, where A is the power within the frequency band of interest at sample *k* and R is mean power of the baseline period. For statistical analysis, results from the time-frequency analysis were averaged over time (300 ms to 500 ms post sign), frequencies, and electrodes, separately for 2 regions of interest (ROI): frontal (AF3, FP1, FPz, FP2, AF4) and posterior (P7, PO7, O1, O2, PO8, P8) ROI. We selected the time window between 300 ms to 500 ms post sign based on the Anguera et al., 2013 study in which the maximum neuronal activity was found within this time window when participants are engaged in Neuroracer multitasking.

### Statistical analyses

In this study, all numerical data are presented as the mean ± the standard error of the mean (SEM). Differences between the stimulation and control groups in terms of behavioral performance as well as neurophysiological measures were assessed by analysis of covariance (ANCOVA) with pre-stimulation data as a covariate and post-stimulation data as a dependent measure. The ANCOVA has been used to assess cognitive training outcomes and it is a suitable approach when the post-intervention measure is not predictable based on pre-intervention measure [35, 36]. As the model assesses the difference in the post-intervention measures after accounting for pre-intervention measures, the results of the analyses reflect the effect of the stimulation, and are not affected by pre-stimulation differences between groups. The difference between stimulation and control runs in terms of the perception of tACS sensation (scaled from 1 to 10) was analyzed with t-tests. Pearson’s correlation was conducted to scrutinize the relationship between multitasking performance and neurophysiological measurements. The normality of the EEG data was assessed with the Kolmogorov—Smirnov) test. The statistical significance threshold was set as p≦0.05.

## Results

All the participants tolerated the repeated runs of tACS well and no one reported any adverse effects.

### tACS effects on multitasking performance

**Fig 2:**
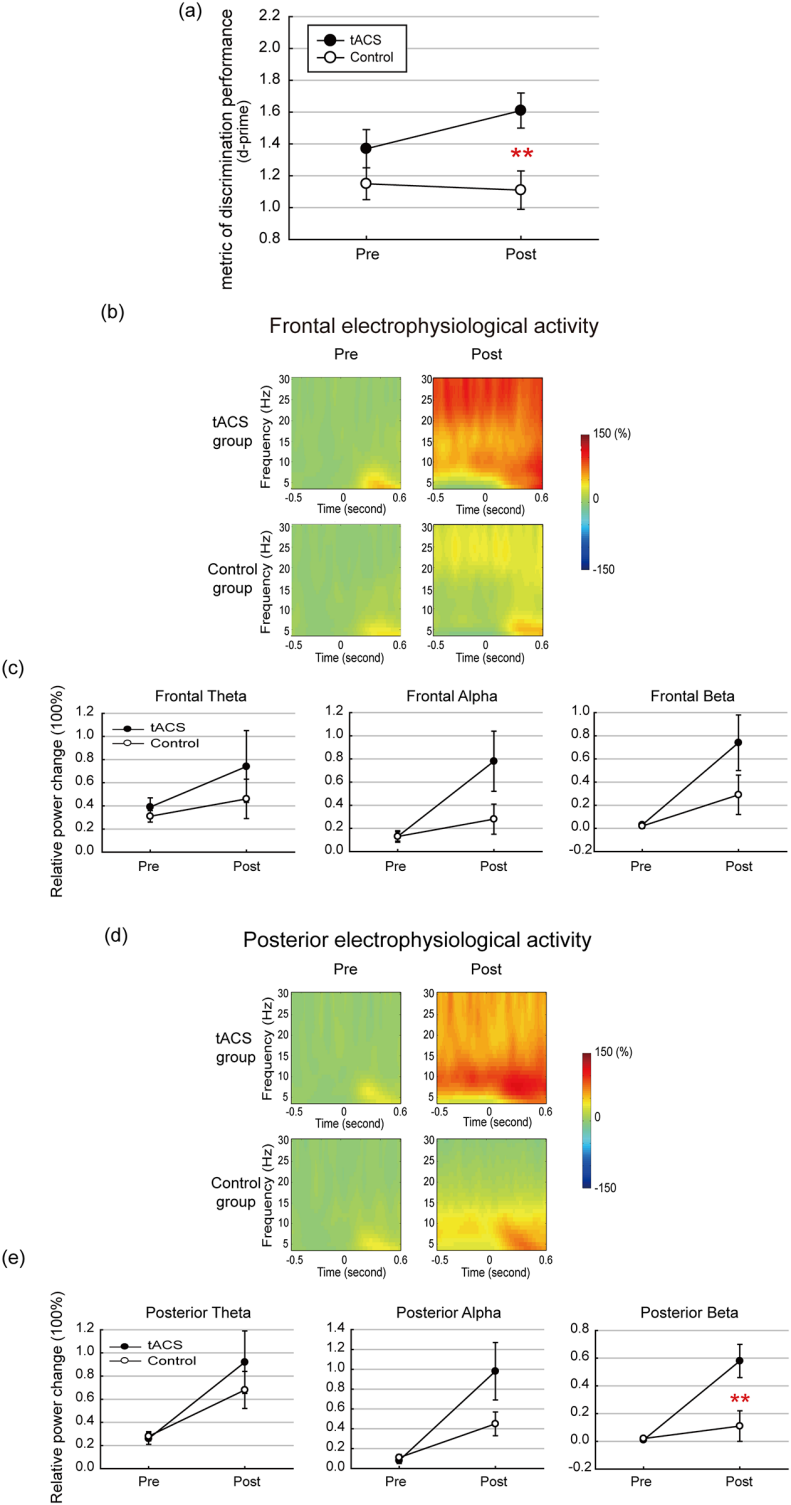
Multitasking performance and neurophysiological activity. (a) Higher multitasking d-prime was observed in tACS group as compared to the control group. (b) and (d) Grand averaged time-frequency representation plots for frontal and posterior electrophysiological activity. (c) and (e) The mean relative power change from pre-stimulation to post-stimulation for frontal and posterior theta, alpha, and beta activity. Error bars represent SEM. **p≦0.01.

There was no significant difference in multitasking performance between the stimulation and active control groups at baseline (t_36_ = 1.30, p = 0.20). An ANCOVA was performed to evaluate differences between the two groups in terms of tACS effects on multitasking performance on Neuroracer over the course of the 90-minute session. There was a significant between group effect of tACS on post-stimulation multitasking performance after accounting for pre-stimulation performance (F_1,35_ = 6.63, p = 0.01, ηp^2^ = 0.16) (Fig 2a). Next, an effect size for d’ was calculated based on the mean and the standard deviation of the post-stimulation performance using Cohen’s d [37]. The difference between the tACS and control groups yielded a strong effect size of 0.96 with 95% confidence interval (CI) ranged between 0.28 and 1.63. Of note, results of two-way repeated measures ANOVA with group (tACS, control) as a between-subject factor and time (pre, post) as a within-subject factor yields comparable results. Detailed pre-stimulation and post-stimulation behavioral results are listed in Table 1. Details of how multitasking performance changed during (i.e., online) and after (i.e., offline) the stimulation are provided in S1 Result, S1 Fig and S1 Table).

**Table 1:** Behavioral and neurophysiological results.

	Group	Pre-stimulation	Post-stimulation
**D-prime**	tACS	1.37 (0.12)	1.61 (0.11)
	Control	1.15 (0.10)	1.11 (0.12)
**Frontal theta**	tACS	0.39 (0.08)	0.74 (0.31)
	Control	0.31 (0.05)	0.46 (0.17)
**Frontal alpha**	tACS	0.13 (0.05)	0.78 (0.26)
	Control	0.13 (0.04)	0.28 (0.13)
**Frontal beta**	tACS	0.03 (0.01)	0.74 (0.24)
	Control	0.02 (0.02)	0.29 (0.17)
**Posterior theta**	tACS	0.26 (0.05)	0.92 (0.27)
	Control	0.28 (0.04)	0.68 (0.16)
**Posterior alpha**	tACS	0.08 (0.03)	0.98 (0.29)
	Control	0.11 (0.03)	0.45 (0.12)
**Posterior beta**	tACS	0.02 (0.01)	0.58 (0.12)
	Control	0.02 (0.01)	0.11 (0.11)

The behavioral results revealed that theta-tACS generated positive effects on multitasking performance, as indicated by the higher post-stimulation d-prime in the tACS group than the control group. In order to examine whether the improved visual discrimination performance in tACS group was achieved at the cost of the visuomotor tracking (driving) task, we analyzed visuomotor tracking performance. The percentage of time the car stayed on the road in the time window from 500 ms before to 1000 ms after the onset of the target signs (green circles) was calculated. The ANCOVA showed no significant differences in tracking performance (F_1,32_ = 1.23, p = 0.27, ηp^2^ = 0.03). These results suggested that improvement of the visual discrimination task in the tACS group was not caused by trade-off in performance between the two tasks.

### tACS effects on neurophysiological measures

Due to the large EEG artifacts induced by tACS, analyses of electrophysiological activity focused only on offline runs (i.e., pre- and post-stimulation). As the nature of the present study was to explore neurophysiological effects of tACS, power changes of oscillatory activity as a function of time were calculated for theta (4-7Hz), alpha (8-12Hz), and beta (13-30Hz) in frontal and posterior regions. ANCOVA analyses were performed to evaluate differences between the stimulation and control groups in terms of tACS effects on these electrophysiological measurements. The Kolmogorov—Smirnov) test revealed that most of the EEG measures are normally distributed (All p>0.05). Only frontal alpha differs significantly from normal distribution (p = 0.004); however, the main results of the current study did not change after removing the outliers in frontal alpha dataset.

Fig 2b depicts grand-averaged time-frequency representation plots for frontal electrophysiological activity. ANCOVAs with pre-stimulation data as a covariate and post-stimulation data as a dependent measure showed no significant differences in frontal theta (F_1,32_ = 0.16, p = 0.68, ηp^2^ = 0.005), alpha (F_1,32_ = 3.24, p = 0.08, ηp^2^ = 0.09) or beta (F_1,32_ = 2.20, p = 0.14, ηp^2^ = 0.06) between the two groups (Fig 2c).

Fig 2d illustrates time-frequency representations from posterior electrophysiological activity. Statistical analyses of posterior electrophysiological activity demonstrated no significant group differences in posterior theta (F_1,32_ = 0.78, p = 0.38, ηp^2^ = 0.02) or alpha (F_1,32_ = 3.35, p = 0.07, ηp^2^ = 0.09) power. However, a significant group difference in posterior beta power was observed (F_1,32_ = 7.66, p = 0.009, ηp^2^ = 0.19) (Fig 2e). A between-group evaluation of the effect size also showed a strong effect size (0.96, 95%CI: 0.27 to 1.67) for post-stimulation posterior beta power (see Table 2 for a summary of the statistical analyses). Results from ANOVAs reveal comparable results (see S2 Table). Detailed pre-stimulation and post-stimulation neurophysiological results are listed in Table 1. Summary of analyses of the 8 electrodes not included in the main analysis is provided in S3 Table.

**Table 2:** Summary of statistical analyses.

	ANCOVA	Cohen’s d (95% CI)
**D-prime**	F(1,35) = 6.63, **p = 0.01**, ηp^2^ = 0.16	0.96 (0.28~1.63)
**Frontal theta**	F(1,32) = 0.16, p = 0.68, ηp^2^ = 0.005	0.26 (-0.40~0.93)
**Frontal alpha**	F(1,32) = 3.24, p = 0.08, ηp^2^ = 0.09	0.59 (-0.08~1.27)
**Frontal beta**	F(1,32) = 2.20, p = 0.14, ηp^2^ = 0.06	0.50 (-0.16~1.18)
**Posterior theta**	F(1,32) = 0.78, p = 0.38, ηp^2^ = 0.02	0.30 (-0.35~0.97)
**Posterior alpha**	F(1,32) = 3.35, p = 0.07, ηp^2^ = 0.09	0.56 (-0.11~1.23)
**Posterior beta**	F(1,32) = 7.66, **p = 0.009**, ηp^2^ = 0.19	0.96 (0.27~1.67)

CI, confidence interval.

Interestingly, the application of tACS affected power in a frequency (i.e. beta) other than the stimulating frequency (i.e. theta). Increased beta activity outside motor-related regions is thought to reflect anticipatory attention processes [38, 39], or more generally, the maintenance of the current cognitive state [40]. In the current study paradigm, increased beta power would serve to maintain sensorimotor representations of the driving task so that attention may be allocated to the appearance of an impending sign for discrimination without altering concurrent driving performance. If this were true, then increased beta power would also be observed in anticipation of sign onset. To test this hypotheses, we conducted a pre-stimulus (-500 ms to stimulus sign onset) time frequency analyses to examine overall power change in posterior beta. ANCOVA analysis with pre-stimulation data as a covariate and post-stimulation data as a dependent measure showed significant group differences in posterior beta power (F_1,32_ = 7.67, p = 0.009, ηp^2^ = 0.19). Between-group evaluation of the effect size also showed a strong effect size (1.00, 95% CI: 0.29 to 1.70) for post-stimulation posterior beta. The result of this pre-stimulus analysis supports the hypothesis that tACS optimized the maintenance of sensorimotor representations during multitasking task.

Of note, the application of theta tACS failed to significantly increase endogenous theta rhythms. Since the current stimulation frequency was fixed at 6 Hz, it may not have targeted the peak theta frequency at the individual level, thus the stimulation effects may be suboptimal and were not able to lead to a significant change in theta power. To test this hypothesis, we split the participants into two groups based on their baseline peak theta band (peak at 6 Hz vs. peak not at 6 Hz). T-tests showed that there was no significant difference in either change of d-prime (post-pre) (t_15_ = 0.93, p = 0.36) or change of frontal theta (t_15_ = 0.37, p = 0.71) between the two groups. Therefore, this possibility to account for the lack of theta effects seems unlikely in the present study.

### Relationship between behavioral and neurophysiological measurements

**Fig 3:**
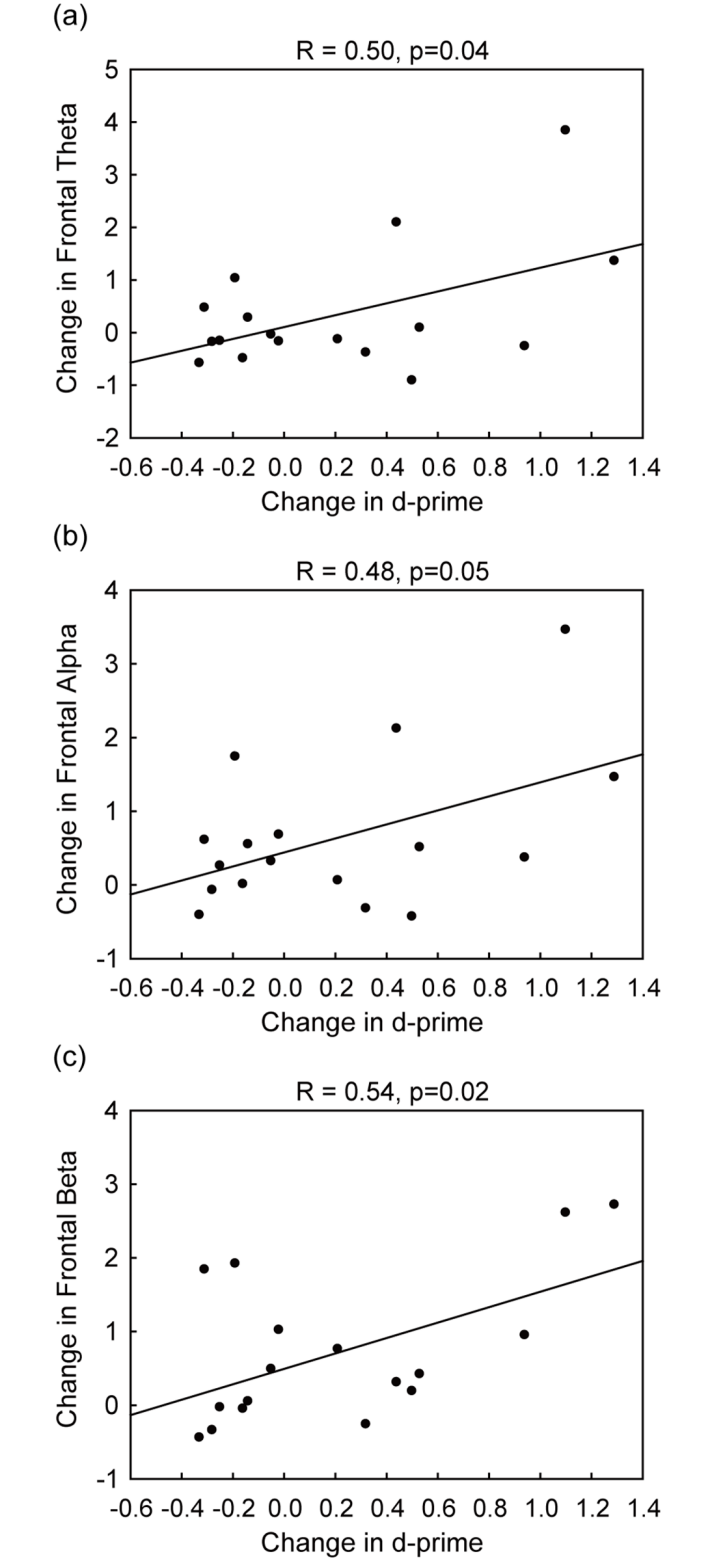
Neurobehavioral correlation for tACS group. Correlation in tACS group between multitasking behavioral gain and changes in (a) frontal theta power (b) frontal alpha power and (c) frontal beta power.

To elucidate the relationship between behavioral and neurophysiological measurements, we performed Pearson’s correlation analyses on changes in oscillatory activity and d-prime. The tACS group demonstrated positive correlations between improvement in multitasking d-prime and enhancement of frontal theta (r = 0.50, p = 0.04), frontal alpha (r = 0.48, p = 0.05) as well as frontal beta power (r = 0.54, p = 0.02) (Fig 3). However, correlations between d’ change and changes in posterior EEG measurements were not significant (all p > 0.15). The control group did not show any significant correlations between performance and frontal or posterior oscillatory power (all p > 0.14).

### Confirmation of participant blinding

Confirming that the control runs was an appropriately blinded manipulation, there were no statistical differences in the perception of stimulation between stimulation and control runs for the stimulation group (all nine measures p > 0.10). Importantly, there were also no statistical differences in the perception of stimulation between the stimulation group during their tACS runs and the corresponding control runs in the control group (all p > 0.25). Other than having participants scale the perception of stimulation from 1 (mild) to 10 (severe), we explicitly asked participants if they know they were in the stimulation or control group. As all of our participants were naïve to tACS studies, most of them thought they were in the stimulation group (including participants in the control group). Only 1 of the participants in the control group thought they were in the control condition. To confirm the task performance was not affected by potential visual phosphenes (i.e. flickering perception of the light) induced by tACS [18, 41], we explicitly asked participants if they saw slight “flickering” during the stimulation runs and none of the participants reported a phosphene effect. Given that phosphenes are most likely when stimulation is at higher frequencies than theta band [42] and none of the participants experienced phosphenes, it seems unlikely that the current results would be confounded by visual phosphenes.

## Discussion

We studied the effects of frontal theta electrical stimulation on multitasking performance, as well as changes in neurophysiological measures associated with tACS effects. The results showed that repeated runs of theta-tACS generates positive effects on multitasking performance accompanied by an offline increase in posterior beta power. Additionally, changes in frontal oscillations positively correlated with changes in multitasking performance. These results suggest that applying frontal theta stimulation increases power in multiple frequencies of brain oscillations related to improved multitasking performance.

### tACS effects on multitasking performance and neurophysiological measurements

The present results indicated that low frequency oscillating (i.e. theta) currents introduced extracranially to the brain modulated higher frequency oscillations (i.e. beta oscillations) in brain regions close to and distant from the stimulation sites. This finding corroborates several previous reports. With intracranial recordings, Ozen et al., (2010) demonstrated that weak slow oscillatory stimulation could affect neurons in widespread cortical areas, including regions distant from the site of stimulation. Notably, a larger fraction of neurons were affected as the stimulation intensity increased [43]. It has been proposed that depending on the intensity of the stimulation, neurons distant from the stimulation site can be activated directly via emphatic coupling or indirectly through synaptic connections [44, 45]. A relatively high current density (318.5 μA/cm^2^) was applied in the present study in comparison to other tACS studies that do not exhibit effects in distant regions or other frequency bands (14.2 μA/cm^2^~28.6 μA/cm^2^) [27, 29, 46]. The stimulation electrodes were centered at F3 and F4 of the 10–20 system to be over bilateral dorsolateral prefrontal cortex (DLPFC) [47]. Since there are abundant structural and functional connections between frontal, parietal and visual cortex to direct top-down modulation of sensory activity that mediate bottom-up information processes [48, 49], the distant neurophysiological effects generated by tACS might be a result of the potentiation of these long-range associative connections.

In contrast to previous studies [29, 50, 51], our results suggest that tACS can affect power in a frequency (i.e. beta) other than the stimulating frequency (i.e. theta). Similar cross-frequency modulation has been reported in several other studies [30–32, 52]. Notably, a recent study showed that by applying theta-tACS over parietal regions, alpha power decreases in multiple regions across the whole brain [31]. Indeed, with a repetitive stimulation protocol (five 5-min stimulation runs with 1-min interval in between) and a high current density (517 μA/cm^2^), Kirov et al., (2009) found a pronounced and widespread power enhancement in theta and beta oscillations after transcranial slow oscillation stimulation at 0.75 Hz. The exact cellular mechanism involved in such a cross-frequency modulation is elusive. However, the similarity between the current findings and the effects observed by Kirov et al., (2009) likewise suggests that repeated runs of oscillatory stimulation with short inter-run-intervals (1-min) and high current density (larger than 300 μA/cm^2^) may result in general functional neuroplastic effects. Neuroplastic changes have been proposed to be responsible for the after-effects of tACS [28, 53], although an online tACS-induced neural entrainment may be a prerequisite for such plasticity [54]. Ali et al., (2013) demonstrated that periodic perturbation (i.e. 3-Hz stimulation) effectively increased the power of higher harmonics of the resonant frequency (i.e. 6 Hz). Furthermore, the range of the affected frequencies broadened as the stimulation intensity increased [30]. Since the current density of the present study is relatively high, the cross-frequency modulation might be a consequence of network entrainment. An important question for future research is to address the frequency specificity and intensity required to yield the observed effects.

To successfully perform a multitasking challenge, several domains of cognitive control abilities are required, such as sustained attention [55], task switching [56], selective attention [57], and inhibitory control [58]. Given that multitasking is a higher-order cognitive process that involves the integration of processes from many domains of cognitive control abilities, a multi-frequency network may exist to support cognitive subcomponents that contribute to the successful multitasking behavior. The interregional cross-frequency modulation induced by tACS may be the result of enhancing multiple cognitive functions subserving multitasking behavior. Therefore, instead of affecting endogenous brain oscillations in a specific frequency manner, tACS may have generated an overall positive effect on multiple active cognitive networks, which may explain the positive correlations between the improvement of multitasking performance and the enhancement of frontal theta, alpha, and beta power.

Another important open question is what do posterior beta power changes represent in this context. Increased beta activity outside motor-related regions is associated with anticipatory attention processes [38, 39], or maintenance of the current cognitive state [40]. In other words, beta activity is associated with the continuation of the sensorimotor cognitive set by helping endogenous top-down influences manage the effect of impending external events [40]. In the current context, increased beta power may serve to maintain sensorimotor representations of the driving task so that attention may be allocated to the appearance of an impending sign for discrimination without changing concurrent driving performance. The result of the pre-stimulus analysis shows that tACS resulted in an anticipatory change in posterior beta power, which supports the hypothesis that tACS optimized the maintenance of sensorimotor representations that contributes to successful multitasking behavior.

Interestingly, the application of theta tACS over bilateral PFC failed to significantly increase endogenous theta rhythms. One explanation is suggested by studies that have indicated stimulation effects are most efficient when the externally applied frequency is at the intrinsic frequency [29]. However, this possibility seems unlikely in the present study according to our analyses based on baseline peak theta band. As effects of transcranial electrical stimulation on electrophysiological activity critically depend upon the prevailing brain-state [27, 59] and because young adults demonstrate relatively high theta power during multitasking [7], a more plausible explanation to account for this finding is that during multitasking, endogenous theta power has reached a maximal level and cannot be further enhanced (i.e. ceiling effect). Since the effects of non-invasive brain stimulation may be graded across different populations from healthy young adults to physiological aging and pathological conditions [60], it is possible that the effect can be only observed in older adults, clinical populations or younger adults with room to improve. Indeed, this interpretation receives some support from the correlations that show participants who were able to increase theta power exhibited the greatest performance improvements.

Regarding the interpretation of the present results, it is important to note that the lack of a frequency control condition means we offer no direct evidence that the observed behavioral and neurophysiological effects are specifically dependent upon theta-tACS. Similarly, without a control site for stimulation, it is unclear whether these effects are specific to PFC stimulation or whether similar effects may be observed by stimulating other cortical regions. This will be the focus of future research.

### Effects of transcranial electrical stimulation on multitasking performance

We have previously demonstrated that anodal tDCS over left DLPFC enhances multitasking performance in the same paradigm as implemented here [10]. The difference between the tDCS and control groups was equivalent to a strong effect size of 0.75 with 95% CI ranged from -0.04 to 1.55. Similarly, the present study showed a strong effect size in post-stimulation multitasking performance (effect size: 0.96; CI: 0.28~1.63). Both studies support the notion that transcranial electrical stimulation is promising for cognitive enhancement. However, the differences in experimental design between the two studies (i.e. type of electrical stimulation (DC vs. AC), current density, and stimulation duration) make it difficult to compare the two studies directly. Also, it is difficult to draw broader conclusion regarding neural mechanisms as tDCS is thought to modulate cortical excitability [61] whereas tACS is thought to influence endogenous brain oscillations [11–13]. Nonetheless, it is still interesting to note that both techniques improved multitask performance, with the use of tACS yielding a slightly larger effect size than tDCS. Additional research will be required to fully control for confounding factors in order to draw stronger conclusions regarding the utility of AC compared to DC stimulation.

## Conclusions

The present results provide evidence that theta-tACS over bilateral PFC improves multitasking performance and modulates related neural oscillations. Our findings suggest that tACS may be a promising tool for cognitive enhancement, as well as raise the possibility of potential therapeutic applications for physiological aging and clinical populations afflicted with cognitive deficits (e.g. Alzheimer’s disease) [62] as the effects outlast the duration of the stimulation. Studies with appropriate frequency and site control conditions will help to unequivocally demonstrate the specificity of stimulation effects. Further research is warranted to determine the impact of longer-term stimulation over the course of days, as well as sustainability of stimulation effects and potential transfer of benefits to other cognitive control abilities.

## Supporting information

S1 Dataset De-identified individual data. (XLSX)

S1 Fig Online and offline effects of tACS on multitasking performance. (DOC)

S1 Result Supplementary Results. (DOC)

S1 Table Detailed online and offline behavioral results. (DOC)

S2 Table Summary of two-way repeated ANOVA with group (tACS, control) as a between-subject factor and time (pre, post) as a within-subject factor. (DOC)

S3 Table Summary of ANCOVA analyses of the 8 electrodes not included in the main analysis. (DOC)
