# Supplementary material for: Enhancement of multitasking performance and neural oscillations by transcranial alternating current stimulation
Source: PLoS One. 2017 May 31;12(5):e0178579. doi: 10.1371/journal.pone.0178579 (PMC5451121; doi:10.1371/journal.pone.0178579)
Supplement: S1 Fig — (DOC) [file pone.0178579.s002.doc]

**S1 Figure. Online and offline effects of tACS on multitasking performance**


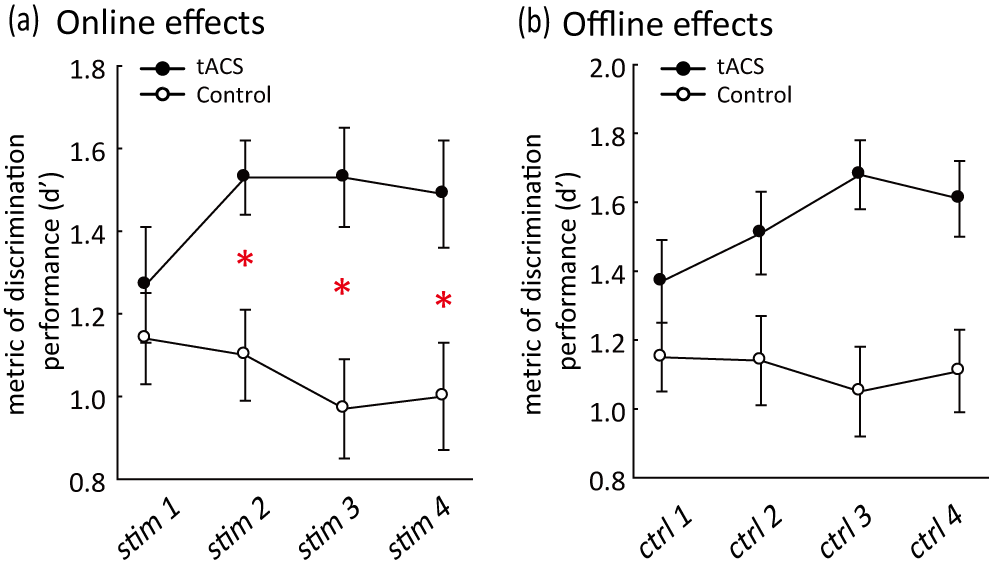


Error bars represent SEM. *p≦0.01.
