# Supplementary material for: Enhancement of multitasking performance and neural oscillations by transcranial alternating current stimulation
Source: PLoS One. 2017 May 31;12(5):e0178579. doi: 10.1371/journal.pone.0178579 (PMC5451121; doi:10.1371/journal.pone.0178579)
Supplement: S1 Result — (DOC) [file pone.0178579.s003.doc]

**S1 Result. Supplementary Results**
 To examine tACS online effects, the data were grouped into four points: stim1

(runs 3+4), stim2 (runs 5+6), stim3 (runs 11+12), and stim4 (runs 13+14). Since the stimulation and control groups had different stimulation conditions from stim1, it is not appropriate to include stim1 as a baseline covariate. Therefore, differences between the two groups were assessed by two-way repeated measures ANOVA with group (tACS, control) as a between-subject factor and time (stim1, stim2, stim3, stim4) as a within-subject factor. A Greenhouse-Geisser correction was applied when appropriate, with t-tests carried out for direct comparisons. The results showed significant group main effect (F(1,36)=6.86, p=0.01, ηp^2^=0.16) and time by group interaction (F(3,108)=3.43, p=0.02, ηp^2^=0.08). No time main effect was observed (F(2.24, 80.84)=0.76, p=0.48, ηp^2^=0.02). Post-hoc tests revealed that the tACS stimulation group showed a higher d’ in stim2 (t_36_=2.71, p=0.01), stim3 (t_36_=3.25, p=0.002) and stim4 (t_36_=2.60, p=0.01) compared to control group (S1 Fig (a); S1 Table). The results suggested that tACS generated online effects on multitasking performance.

To provide further information about tACS effects on multitasking performance, offline behavioral data were grouped into four points: ctrl1 (runs 1+2), ctrl2 (runs 7+8), ctrl3 (runs 9+10), and ctrl4 (runs 15+16). An ANCOVA with group (tACS, control) and time (ctrl2, ctrl 3, ctrl 4) as factors was conducted with ctrl1 as a baseline covariate. No time main effect (F(2,70)=0.07, p=0.92, ηp^2^=0.002) or time by group interaction (F(2,70)=1.32, p=0.27, ηp^2^=0.03) was observed. However, significant group main effect was found (F(1,35)=9.10, p=0.005, ηp^2^=0.20) (S1 Fig (b); S1 Table). The results of this additional analysis are in line with Fig 2a, which shows that theta-tACS resulted in multitasking performance difference between the two groups.
