# Supplementary material for: Enhancement of multitasking performance and neural oscillations by transcranial alternating current stimulation
Source: PLoS One. 2017 May 31;12(5):e0178579. doi: 10.1371/journal.pone.0178579 (PMC5451121; doi:10.1371/journal.pone.0178579)
Supplement: S1 Table — (DOC) [file pone.0178579.s004.doc]

**S1 Table. Detailed online and offline behavioral results**

|  | **stim1** | **stim2** | **stim3** | **stim4** | **ctrl1** | **ctrl2** | **ctrl3** | **ctrl4** |
| --- | --- | --- | --- | --- | --- | --- | --- | --- |
| **tACS** | **1.27 (0.14)** | **1.53 (0.09)** | **1.53 (0.12)** | **1.49 (0.13)** | **1.37 (0.12)** | **1.51 (0.12)** | **1.68 (0.10)** | **1.61 (0.11)** |
| **Control** | **1.14 (0.11)** | **1.10 (0.11)** | **0.97 (0.12)** | **1.00 (0.13)** | **1.15 (0.10)** | **1.14 (0.13)** | **1.01 (0.13)** | **1.11 (0.12)** |
