# Supplementary material for: Enhancement of multitasking performance and neural oscillations by transcranial alternating current stimulation
Source: PLoS One. 2017 May 31;12(5):e0178579. doi: 10.1371/journal.pone.0178579 (PMC5451121; doi:10.1371/journal.pone.0178579)
Supplement: S2 Table — (DOC) [file pone.0178579.s005.doc]

**S2 Table. Summary of two-way repeated ANOVA with group (tACS, control) as a between-subject factor and time (pre, post) as a within-subject factor**

|  | **Source (df)** | **F** | **p-value** | **ηp^2^** |
| --- | --- | --- | --- | --- |
| **D-prime** | **Time (1,36)** | **1.51** | **0.22** | **0.04** |
|  | **Group (1,36)** | **5.82** | **0.02** | **0.13** |
|  | **[T]x[G] (1,36)** | **3.30** | **0.06** | **0.08** |
| **Frontal theta** | **Time (1,33)** | **2.41** | **0.13** | **0.06** |
|  | **Group (1,33)** | **0.74** | **0.39** | **0.02** |
|  | **[T]x[G] (1,33)** | **0.37** | **0.54** | **0.01** |
| **Frontal alpha** | **Time (1,33)** | **8.79** | **0.006** | **0.21** |
|  | **Group (1,33)** | **2.47** | **0.12** | **0.07** |
|  | **[T]x[G] (1,33)** | **3.31** | **0.07** | **0.09** |
| **Fontal beta** | **Time (1,33)** | **10.82** | **0.002** | **0.24** |
|  | **Group (1,33)** | **2.25** | **0.14** | **0.06** |
|  | **[T]x[G] (1,33)** | **2.28** | **0.14** | **0.06** |
| **Posterior theta** | **Time (1,33)** | **12.42** | **0.001** | **0.27** |
|  | **Group (1,33)** | **0.40** | **0.52** | **0.01** |
|  | **[T]x[G] (1,33)** | **0.79** | **0.38** | **0.02** |
| **Posterior alpha** | **Time (1,33)** | **15.88** | **<0.001** | **0.32** |
|  | **Group (1,33)** | **2.10** | **0.15** | **0.06** |
|  | **[T]x[G] (1,33)** | **3.24** | **0.08** | **0.09** |
| **Posterior beta** | **Time (1,33)** | **15.67** | **<0.001** | **0.32** |
|  | **Group (1,33)** | **7.58** | **0.01** | **0.18** |
|  | **[T]x[G] (1,33)** | **7.99** | **0.008** | **0.19** |

**[T] x [G], time by group interaction.**
