# Supplementary material for: Enhancement of multitasking performance and neural oscillations by transcranial alternating current stimulation
Source: PLoS One. 2017 May 31;12(5):e0178579. doi: 10.1371/journal.pone.0178579 (PMC5451121; doi:10.1371/journal.pone.0178579)
Supplement: S3 Table — (DOC) [file pone.0178579.s006.doc]

**S3 Table. Summary of ANCOVA analyses of the 8 electrodes not included in the main analysis**

The 8 electrodes were grouped into three regions of interest based on their location: frontal (AFz, Fz, F7, F8), central (C3, Cz, C4) and posterior (Pz). ANCOVAs with pre-stimulation data as a covariate and post-stimulation data as a dependent measure showed no significant differences between the two groups.

|  | **F-value** | **p-value** | **ηp^2^** |
| --- | --- | --- | --- |
| **Frontal theta** | **F(1,32)=0.23** | **0.63** | **0.007** |
| **Frontal alpha** | **F(1,32)=1.40** | **0.24** | **0.04** |
| **Frontal beta** | **F(1,32)=2.42** | **0.12** | **0.07** |
| **Central theta** | **F(1,32)=1.24** | **0.27** | **0.03** |
| **Central alpha** | **F(1,32)=3.00** | **0.09** | **0.08** |
| **Central beta** | **F(1,32)=2.40** | **0.10** | **0.07** |
| **Posterior theta** | **F(1,32)=0.70** | **0.40** | **0.02** |
| **Posterior alpha** | **F(1,32)=2.70** | **0.11** | **0.07** |
| **Posterior beta** | **F(1,32)=1.89** | **0.17** | **0.05** |
